# Altered Brain Function in Cerebral Small Vessel Disease Patients With Gait Disorders: A Resting-State Functional MRI Study

**DOI:** 10.3389/fnagi.2020.00234

**Published:** 2020-08-31

**Authors:** Xia Zhou, Chao Zhang, Linlin Li, Yimei Zhang, Wei Zhang, Wenwen Yin, Xianfeng Yu, Xiaoqun Zhu, Yinfeng Qian, Zhongwu Sun

**Affiliations:** ^1^Department of Neurology, The First Affiliated Hospital of Anhui Medical University, Hefei, China; ^2^Department of Neurology, The First Affiliated Hospital of University of Science and Technology of China, Hefei, China; ^3^Department of Radiology, The First Affiliated Hospital of Anhui Medical University, Hefei, China

**Keywords:** cerebral small vessel disease, gait disorder, ALFF/fALFF, functional connectivity, supplementary motor area (SMA)

## Abstract

Gait disturbances are important clinical features of cerebral small vessel disease (CSVD) that increase the risk of falls and disability. Brain structural alterations and gait disturbances in CSVD patients have been well demonstrated. However, intrinsic resting cerebral function patterns in CSVD patients with gait disorders remain largely unknown. Fifty-eight CSVD patients were enrolled in our studies and categorized into the gait disorder group (CSVD-GD, *n* = 29) and no-gait disorder group (CSVD-NGD, *n* = 29) based on a gait examination. Gait was quantitatively assessed with the Timed Up and Go test and the intelligent device for energy expenditure and activity (IDEEA). Functional MRI and fractional amplitude of low-frequency fluctuation (fALFF) analyses were employed to explore local intrinsic neural oscillation alterations. Functional connectivity based on fALFF results was calculated to detect the potential changes in remote connectivity. Compared with the CSVD-NGD group, the CSVD-GD group showed decreased fALFF in regions mainly located in the sensorimotor network and frontoparietal network, such as the left supplementary motor area (SMA.L) and the left superior parietal gyrus, and increased fALFF in the right inferior frontal gyrus (orbital part), the left caudate, and the left precuneus. Moreover, the CSVD-GD patients exhibited lower connectivity between the SMA.L and temporal lobe, which was related to gait speed. The fALFF value of the SMA.L was associated with cadence. This study highlights the regional and network interaction abnormalities of the SMA in CSVD patients with gait disturbances. These findings could provide further insight into the neural mechanisms of gait disturbances in CSVD.

## Introduction

Gait disturbances are quite common in elderly adults and are associated with an increased risk of falls, disability, and mortality (van der Holst et al., 2016). Normal gait control involves both the motor system and cognition control system (Rosano et al., 2012). The effect of peripheral nervous system factors, such as sarcopenia or plantar cutaneous sensory on gait stability, has received ample attention in recent years (Kwon et al., 2019; Navarro-Peternella et al., 2019); however, research on the influence of central nervous system factors is still limited. Recently, an extensive body of evidence has focused on the contributing role of cerebral small vessel disease (CSVD) on gait impairments in elderly individuals (Pinter et al., 2017; Loos et al., 2018; van der Holst et al., 2018). White matter hyperintensities (WMHs) and lacunar infarcts (LAs), two major imaging markers of CSVD, have been suggested to be correlated with motor impairments in balance and gait function, even after adjusting for a range of confounding covariates (Smith et al., 2015). Cognition impairment is the most striking symptom of CSVD that attracts much attention from patients. However, subcortical syndromes, such as gait disorders, tend to be neglected because of their insidious onset. Certainly, not all individuals with CSVD eventually experience gait disturbances, although CSVD has been recognized as a possible cause of gait impairment (Su et al., 2018). In other words, the motor impairments associated with CSVD cannot be fully interpreted by WMHs or LAs on MRI. Therefore, a better understanding of the characteristics of gait disturbances and exploring the potential pathophysiological mechanisms of gait disorders in CSVD are critical scientific endeavors that will eventually help to achieve further advances in the therapy of gait disorders.

In recent years, a number of studies have tried to identify structural changes in CSVD patients with gait disorders (de Laat et al., 2011). These studies have shown that widespread structural alterations were exhibited in CSVD patients with gait disturbances, including white matter integrity loss, gray matter atrophy, and cortical thinning. Moreover, loss of microstructural integrity not only within the WMH area but also within the normal-appearing white matter (NAWM) contributes to gait impairments (Rosario et al., 2016). However, very few studies have directly examined functional alteration patterns. Resting-state functional MRI (rs-fMRI) is a potentially powerful approach to investigate brain functional alterations with its unique advantage of not requiring ongoing activity (Fox and Raichle, 2007). The assessment of amplitude of low-frequency fluctuation (ALFF), one of the methods based on rs-fMRI, has been widely applied to probe regional spontaneous brain activity by measuring the square root of the power spectrum in the low-frequency range (Zuo et al., 2010). However, as it is difficult to eliminate physiological noise, the fractional amplitude of low-frequency fluctuation (fALFF) technique has been recommended and calculates the ratio of the low-frequency power spectrum to that of the entire frequency range (Zou et al., 2008), and this method has been used to explore regional spontaneous brain activity in several diseases, such as Parkinson's disease (PD) (Shen et al., 2018) and Alzheimer's disease (AD) (Yang et al., 2018). Functional connectivity (FC) provides additional information by measuring levels of integrated local activity across distant regions and can have utility for advancing our understanding of dysfunctions in integrated brain networks involved in gait disorders associated with CSVD.

Therefore, the aim of the present study was to identify patterns of functional alterations in CSVD patients with gait disturbances using both fALFF and seed-based FC analyses. We hypothesized that altered resting function would not be limited to regional brain areas, but also observed in remote areas or networks that have also been implicated in gait problems in CSVD patients.

## Materials and Methods

### Subjects

Sixty-five right-handed consecutive CSVD patients were enrolled in our study from the First Affiliated Hospital of Anhui Medical University. CSVD was defined as subcortical white matter hyperintensity with a Fazekas scale score ≥2 (at least 2 on the Fazekas scale for either deep or periventricular WMH) or LAs on T2-weighted images and/or fluid-attenuated inversion recovery (FLAIR) images, without any acute stroke appearances in diffusion-weighted imaging, according to the commonly applied criterion (Takakusaki, 2017). WMHs were assessed using the Fazekas scale on the FLAIR sequence (Fazekas et al., 1987). LAs were defined as focal lesions from 3 to 15 mm with cerebral spinal fluid-like signal on FLAIR or T2-weighted images. We also assessed the WMH volume semi-automatically using volumetric measurements and calculated the number of LAs. In brief, lesion load of white matter measurements was performed on FLAIR images by a trained operator (L.L.). All lesion areas were outlined on the computer image and automatically computed by the MRIcro and ITK-SNAP software (Chen et al., 2018).

The exclusion criteria for the CSVD patients included the following: WMH resulting from non-vascular diseases, such as poisoning, multiple sclerosis, encephalitis, and infection; a history of known stroke, head injury, Parkinson's disease, epilepsy, major depression, or other neurological or psychiatric illness; severe visuospatial deficits, hearing impairments, or language disorders; *in vivo* dentures or metallic stents; serious orthopedic diseases, such as osteoarthritis, fracture, congenital malformation, amputation, and arthroplasty of the lower limbs.

The institutional review board of the First Affiliated Hospital of Anhui Medical University Subcommittee on Human Studies approved the study, and informed written consent was obtained from the participants.

### Cognitive and Neuropsychological Assessments

A series of neuropsychological assessments was performed by a trained neuropsychological technician within 1 week after the MRI scan, including the Mini-Mental State Examination (MMSE), Cambridge Cognitive Examination–Chinese version (CAMCOG-C), Geriatric Depression Scale (GDS), Activities of Daily Living Scale (ADL), and Stroop test (Stroop-1: dot; Stroop-2: characters; Stroop-3: color) to evaluate global cognition and functions of episodic memory, attention, psychomotor speed, executive function, visuospatial skills, and emotion. The administration of the battery took between 1.5 and 2.0 h.

### Gait Analysis and Patient Classification

#### Timed Up and Go (TUG) Test and Berg Balance Scale (BBS)

The TUG test (Mathias et al., 1986) is a classic and simple test to assess mobility and walking by measuring the time a person takes to rise from a standard chair without using armrests, walk 3 m at a usual pace, turn around, walk back to the chair, and finally sit down again, and has been used in the assessment of patients with several diseases, such as PD (Sato et al., 2019). The TUG test identified the fall risk among the elderly population at high sensitivity (87%) and specificity (87%) with 15 s as the threshold (Shumway-Cook et al., 2000). In our study, all participants were instructed to take the TUG test twice, and the mean time of the two trials was calculated as the result. In the present study, the CSVD patients were divided into the gait disturbance group (CSVD-GD; TUG test ≥15 s) and no-gait disturbance group (CSVD-NGD; TUG test <15 s).

To obtain more information, the TUG dual task (TUG-2) and BBS test were also performed. The former was executed by extracting all the numbers containing 7 from 1 to 100 (such as 7, 17, 27…) during the walking process and calculating the time and the number of errors. The BBS test was used to measure balance function and included 14 balance tests ranging from simple tasks (such as transfer, unsupported standing, sitting to standing) to complex tasks (such as turning 360°, standing on one leg). Each item was graded on a 5-point scale (0–4), in which grade 0 indicated that the motion could not be performed, and grade 4 indicated that the individual completed the specified task, for a total of 56 points (14 × 4).

#### Intelligent Device for Energy Expenditure and Activity (IDEEA)

The IDEEA (MiniSun, LLC, Fresno, CA, USA), a portable and useful instrument for recording and analyzing physical activities and gait, was used to capture gait parameters (sampling frequency 64 Hz). The IDEEA has been shown to have excellent test–retest reliability and validity (Gorelick et al., 2009). It was performed with seven tiny accelerometers/inclinometers (1.4 × 1.1 × 0.3 cm^3^) fixed to the skin by medical tape. Each participant was asked to walk for 30 m at a comfortable walking speed with IDEEA, and the gait parameters were collected by the device at the same time, which were subsequently transferred to a personal computer for data analysis. To measure steady-state walking, spatiotemporal parameters from only the middle 20 strides, including gait speed, cadence, and stride length, single support duration, and double support duration, were analyzed using IDEEA software.

#### MRI Acquisition

MRI data were obtained using a 3.0 Tesla GE Signa HDxt MRI scanner (GE, Milwaukee, WI, USA) equipped with an eight-channel head coil. All participants were instructed to keep their eyes closed without falling asleep and to think as little as possible during the scan. To minimize head movement and eliminate scanner noise, foam padding and ear plugs were used. The resting-state data were obtained using echo-planar imaging (EPI) at 2-s intervals for a total of 8 min, which comprised 240 contiguous EPI whole-brain functional volumes with the following parameters: repetition time (TR) = 2 s; echo time (TE) = 30 ms; field of view (FOV) = 240 × 240 mm^2^; flip angle (FA) = 80; matrix size = 64 × 64; slice thickness = 4 mm; gap = 0.6 mm. Three-dimensional (3D) T1-weighted images were acquired using a spoiled gradient recalled echo sequence with the following parameters: TR = 9.5 ms; TE = 3.9 ms; FA = 20; FOV = 256 × 256 mm^2^; matrix size = 512 × 512. The following structural brain sequences were also acquired: T2-weighted spin echo (TR = 3,500 ms; TE = 85 ms; echo train length = 15; FA = 90; 22 contiguous, 5-mm-thick, axial slices; matrix size = 512 × 512; and FOV = 230 × 184 mm^2^); FLAIR (TR = 11 s; TE = 120 ms; FA = 90; 22 contiguous, 5-mm-thick, axial slices; matrix size = 512 × 512; and FOV = 230 × 230 mm^2^).

#### Image Processing and Analysis

The rs-fMRI data were preprocessed using Data Processing and Analysis for (Resting-State) Brain Imaging (DPABI) software (Yan et al., 2016) (http://www.restfmri.net) running in MATLAB (Mathworks, Natick, MA, USA). The process comprised removal of the first 10 EPI time points to ensure steady state and allow the participant to acclimate to the scanning environment, slice timing to correct for differences in image acquisition time between slices, image realignment for head motion correction, nuisance covariates regression (the estimated motion parameters based on the Friston-24 model, the cerebrospinal fluid signal, and the white matter signal), spatial normalization to the Montreal Neurological Institute (MNI) by diffeomorphic anatomical registration through exponentiated lie algebra (DARTEL) and resampled at a resolution of 3 × 3 × 3 mm^3^, spatial smoothing with 6-mm full-width at half-maximum Gaussian kernel, detrending to remove the linear trends, and band-pass filtering (0.01–0.08 Hz). During the processing, head motion of more than 2.5 mm maximum translation in any direction of x, y, or z or 2.5° of maximal rotation throughout the course of scanning were excluded (three CSVD-GD patients and four CSVD-NGD patients). We also calculated framewise displacement (FD Jenkinson), which indexes the volume-to-volume changes in head position. No significant group differences were found in FD Jenkinson between the CSVD-GD patients and CSVD-NGD patients (*t* = −0.383, *p* = 0.703).

ALFF analysis was also carried out using DPABI software. A fast-Fourier transform (FFT) algorithm was used to transform the time series for each voxel into frequency domain data. ALFF was calculated by obtaining the square root of the power spectrum from 0.01 to 0.08 Hz, and then the ratio low-frequency fluctuation amplitude (fALFF) was obtained by dividing the ALFF of each voxel by the average over the entire frequency range. fALFF values were converted to Z score.

To explore whether brain regions with fALFF alterations also exhibited FC changes, the peak points of the regions with significant group differences in fALFF were selected as seeds for FC analyses. The time courses of the seed regions and all other brain voxels were extracted for estimating alterations in connectivity. Pearson's correlation coefficients were computed between the mean signal change in the seed and all other voxels across the brain. In addition, Fisher's r to Z-transformation was applied to improve the normality of the correlation coefficients.

#### Voxel-Based Morphometry (VBM)

To clarify whether there were structural differences between the two groups, VBM (http://dbm.neuro.uni-jena.de/vbm8/) and Computational Anatomy Toolbox (CAT12; http://www.neuro.uni-jena.de/cat12/CAT12-Manual.pdf) with SPM12 were used for gray matter analyses. First, 3D T1-weighted images were segmented into gray matter, white matter, and cerebrospinal fluid. Then, high-dimensional DARTEL was used to normalize the gray matter maps to the MNI standard space. The modulated spatial normalized gray matter image was smoothed with an 8-mm FWHM kernel. In addition, to evaluate whether functional alterations were contributed to gray matter atrophy, we extracted the regional gray matter volume (GMV) for the regions of interest based on the results of the fALFF and FC analyses by REST (http://www.restfmri.net/forum/REST_V1.8) software.

#### Statistical Analysis

The Statistical Package of Social Science (IBM SPSS Statistics 22, SPSS Inc., Chicago, IL, USA) was used for *t*-tests, χ^2^-tests, nonparametric analyses, and correlation analyses. The continuous data are presented as the mean ± SD for normally distributed variables and as the median (P_25_, P_75_) for the skew-distributed continuous variables. We also used SPM 8 and DPABI software for statistical analyses for fALFF and FC.

A general linear model–based approach was used to detect significant between-group differences in fALFF using SPM 8. Age, sex, and education were included as covariates. For the FC analysis, a one-sample *t*-test was performed on each z-value map to examine functional connectivity in the two groups. Two-sample *t*-tests were used to compare the z values in each voxel of the above groups. Multiple comparisons were corrected using the cluster-level false discovery rate (FDR) method, resulting in a cluster defining threshold of *p* = 0.001 and a corrected cluster significance of *p* < 0.05. To determine the relationship between z values and clinical measures, Pearson's correlation analyses were performed in all CSVD patients (*p* < 0.05). The results are presented using xjview and BrainNet viewer.

## Results

### Demographic, Clinical, and Neuropsychological Results

The socio-demographic and clinical data for each group are shown in Table 1. No significant differences in age, sex, educational levels, or body mass index (BMI) were found between the CSVD-GD group and CSVD-NGD group. Lower levels of gait speed, cadence, and step length were observed in the CSVD-GD group. Moreover, the CSVD-GD group exhibited longer cycle duration and single support time, but no significant differences in double support time were found.

**Table 1:** Demographic, clinical, and neuropsychological test data.

	**CSVD-GD**	**CSVD-NGD**	***P*-value**
N	29	29	
Age (years)	68.66 ± 6.46	65.45 ± 9.79	0.147
Sex (male/female)	15/14	13/16	0.599
Years of education	9.13 ± 3.90	11.35 ± 4.60	0.053
BMI	24.17 ± 3.35	24.18 ± 3.10	0.995
MMSE	25.14 ± 3.24	25.69 ± 3.32	0.525
CAMCOG-C	77.58 ± 12.55	82.48 ± 11.97	0.134
Orientation	9(8,10)	9(9,10)	0.530
Languages	24(22,27)	27(23,28)	0.064
Memory	16.10 ± 4.79	17.03 ± 5.12	0.525
Attention	7(5,7)	6(5,7)	0.868
Praxis	10(8,12)	11(9,12)	0.148
Calculation	2(2,2)	2(2,2)	0.643
Abstraction	5.76 ± 1.94	5.83 ± 1.93	0.892
Perception	7.24 ± 1.43	7.34 ± 1.49	0.789
ADL	20(20,22.25)	20(20,21)	0.993
GDS	7(3.5,12)	5(3,10)	0.374
Stroop-1	30.65 ± 9.61	23.62 ± 7.35	**0.003**
Stroop-2	35.32 ± 10.70	29.94 ± 10.47	0.058
Stroop-3	48.74 ± 19.64	44.01 ± 14.58	0.302
Enlarged perivascular spaces, no. (%)	5 (17.24%)	6 (20.69%)	0.738
LAs (no.)	7.45 ± 4.01	5.62 ± 3.35	0.065
WMHs (mm^3^)	6.13(2.21,6.13)	3.53(1.46,8.74)	0.266
TUG(s)	17.51(15.48,18.73)	11.18(10.42,11.68)	**<0.001**
TUG-2(s)	17.14 ± 3.94	12.19 ± 2.28	**<0.001**
BBS	53(51.75,56)	55(53,56)	0.483
Step length (m)	0.50 ± 0.08	0.55 ± 0.08	**0.007**
Gait speed (m/s)	0.85 ± 0.18	1.02 ± 0.18	**0.001**
Cadence (steps/min)	101.71 ± 10.41	108.68 ± 10.14	**0.012**
Step duration (s)	1.17 ± 0.10	1.10 ± 0.087	**0.004**
Cycle duration (s)	1.17(1.11,1.21)	1.1(1.05,1.17)	**0.006**
Single support time (%)	38.61(37.51,39.98)	37.42(36.63,38.81)	**0.014**
Double support time (%)	25.64(24.18,26.71)	25.24(23.86,26.12)	0.423

*BMI, body mass index; MMSE, Mini-Mental State Examination; CAMCOG-C, Cambridge Cognitive Examination–Chinese version; ADL, Activities of Daily Living Scale; GDS, Geriatric Depression Scale; LA, lacunar infarct; WMH, white matter hyperintensity; TUG, Timed Up and Go; TUG-2, TUG dual task; BBS, Berg Balance Scale*.

Furthermore, the CSVD-GD group exhibited significantly lower executive function scores, as indicated by the Stroop test. No significant differences were found in the CAMCOG-C, MMSE, GDS, and ADL scores. In addition, the differences between the two groups in the number of LAs and the volume of WMHs did not reach statistical significance.

### fALFF Analysis

**Figure 1:**
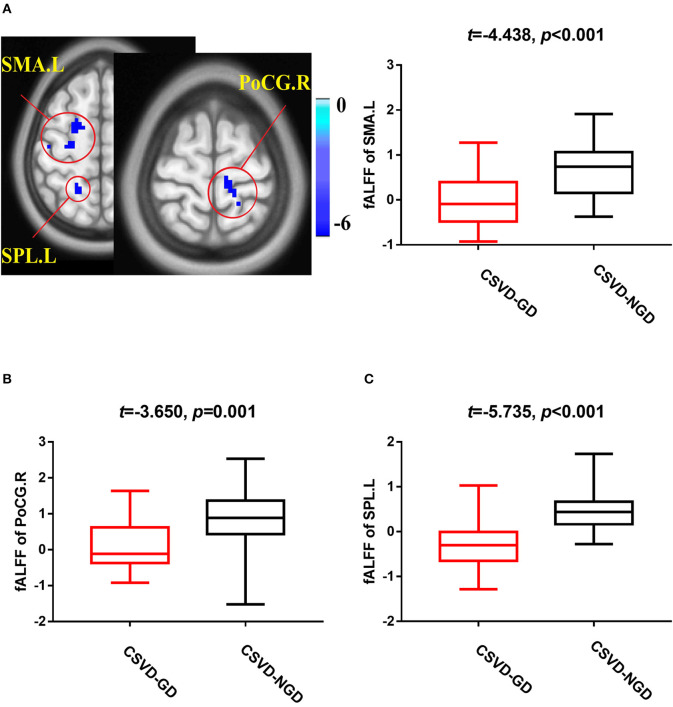
Brain views showing decreased fractional amplitude of low-frequency fluctuations (fALFF) in the SMA.L **(A)**, PoCG.R **(B)**, and SPL.L **(C)** in the CSVD-GD patients compared with the CSVD-NGD patients (*p* < 0.05, cluster-level FDR corrected). The color bar indicates the T score. SMA.L, left supplementary motor area; PoCG.R, right postcentral gyrus; SPL.L, left superior parietal lobe; fALFF, fractional amplitude of low-frequency fluctuations.

**Figure 2:**
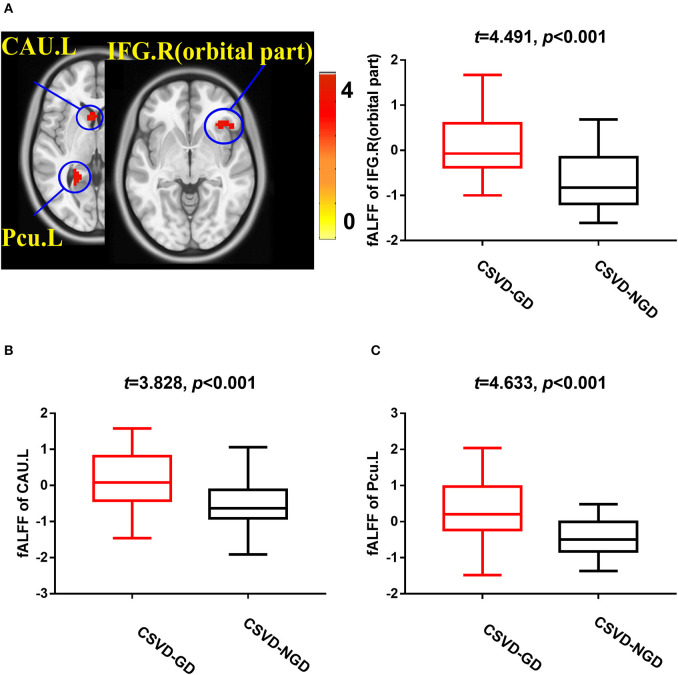
Brain views showing increased fALFF in the IFG.R, orbital part **(A)**, CAU.L **(B)**, and Pcu.L **(C)** in the CSVD-GD patients compared with the CSVD-NGD patients (*p* < 0.05, cluster-level FDR corrected). The color bar indicates the T score. IFG.R, orbital part., right orbital inferior frontal gyrus; CAU.L, left caudate; Pcu.L, left precuneus; fALFF, fractional amplitude of low-frequency fluctuations.

After adjusting for age, sex, and education level, the CSVD-GD group, compared with the CSVD-NGD group, showed a significant fALFF decrease in the left supplementary motor area (SMA.L) (−0.028 ± 0.583 vs. 0.663 ± 0.602, respectively; *t* = −4.438, *p* < 0.001), the left superior parietal lobe (SPL.L) (−0.248 ± 0.509 vs. 0.498 ± 0.481; *t* = −5.735, *p* < 0.001), and the right postcentral gyrus (PoCG.R) (−0.098 ± 0.626 vs. 0.816 ± 0.853; *t* = −3.650, *p*=0.001) (Figure 1), while the fALFF value increased in the right orbital inferior frontal gyrus (IFG.R, orbital part) (0.146 ± 0.707 vs. −0.656 ± 0.651; *t* = 4.491, *p* < 0.001), the left caudate (CAU.L) (0.165 ± 0.781 vs. −0.557 ± 0.677; *t* = 3.828, *p* < 0.001), and the left precuneus (Pcu.L) (0.336 ± 0.779 vs. −0.463 ± 0.505; *t* = 4.633, *p* < 0.001) (Figure 2). These results are summarized in Table 2.

**Table 2:** Brain clusters showing significant differences in the fractional amplitude of low-frequency fluctuations (fALFF) (*p* < 0.05, FDR corrected).

	**Brain regions (AAL)**	**Cluster size (voxels)**	**Peak voxel coordinate—MNI coordinates**			***T*-value**
			**x**	**y**	**z**	
**Decreased regions**						
	SMA.L	108	−18	−3	63	−6.125
	PoCG.R	22	18	−42	72	−4.305
	SPL.L	22	−24	−54	48	−4.179
**Increased regions**						
	IFG.R(Orbital part)	57	45	27	−3	4.443
	CAU.L	28	−6	15	3	4.595
	Pcu.L	21	−24	−54	0	4.615

*AAL, anatomical automatic labeling; MNI, Montreal Neurological Institute; SMA.L, left supplementary motor area; PoCG.R, right postcentral gyrus; SPL.L, left superior parietal lobe; IFG.R(Orbital part), right orbital inferior frontal gyrus; CAU.L, left caudate; Pcu.L, left precuneus*.

### Seed-Based Functional Connectivity Analysis

**Figure 3:**
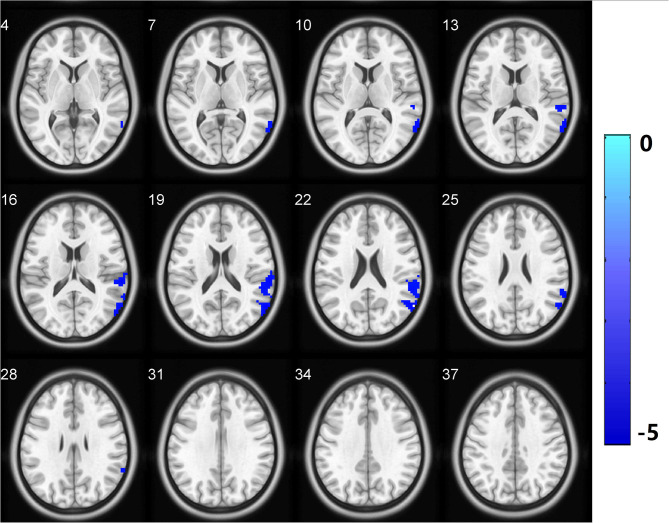
Functional connectivity analysis based on fALFF differences revealed significantly decreased functional connectivity in the LTC.R with the left supplementary motor area as the region of interest in the CSVD-GD patients relative to the controls. All comparisons were performed using two-sample *t*-tests (*p* < 0.05, cluster-level FDR corrected). LTC.R, right lateral temporal cortex. The color bar indicates the T score. fALFF, fractional amplitude of low-frequency fluctuations.

Brain regions with significant group differences in fALFF (i.e., the SMA.L, SPL.L, PoCG.R, IFG.R, orbital part, CAU.L, and Pcu.L) were selected as seeds in the voxel-based FC analysis. The CSVD-GD group, compared with the CSVD-NGD group, showed significantly decreased FC between the left SMA and the right lateral temporal cortex (LTC.R) (−0.072 ± 0.161 vs. 0.142 ± 0.179, respectively, *t* = −4.789, *p* < 0.001), including the right middle temporal gyrus, the supramarginal gyrus and the superior temporal gyrus (*p* < 0.05, cluster-level FDR corrected; minimum cluster size = 213 voxels). The peak voxel coordinates on MNI were *x* = 60, *y* = −63, *z* = 21 and the *T*-value was −5.262 (Figure 3). No significant difference in FC was found in other regions.

### VBM Analysis

There was no significant difference of total GMV between the CSVD-GD and CSVD-NGD patients (540.76 ± 54.37 cm^3^ vs. 550.00 ± 44.09 cm^3^, respectively; *t* = −0.711, *p* = 0.480). In addition, further analysis of regional GMV based on the results of the fALFF and FC analyses did not reveal any differences in local GMV between the two groups, using a significance level of *p* < 0.05, FDR corrected for multiple comparisons (*p* > 0.05).

### Correlation Analysis Results

**Figure 4:**
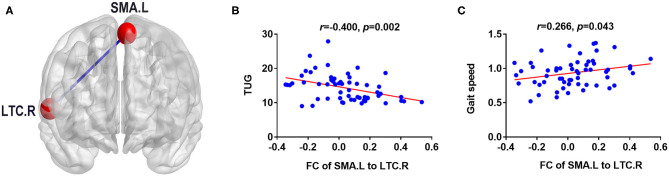
Correlation results between FC (SMA.L-LTC.R) and clinical variables (*p* < 0.05, cluster-level FDR corrected). **(A)** FC analysis with the left supplementary motor area as the seed. **(B)** Negative correlation between FC (SMA.L-LTC.R) and TUG. **(C)** Positive correlation between FC (SMA.L-LTC.R) and gait speed. FC, functional connectivity; LTC.R, right lateral temporal cortex; SMA.L, left supplementary motor area; TUG, Timed Up and Go test.

**Figure 5:**
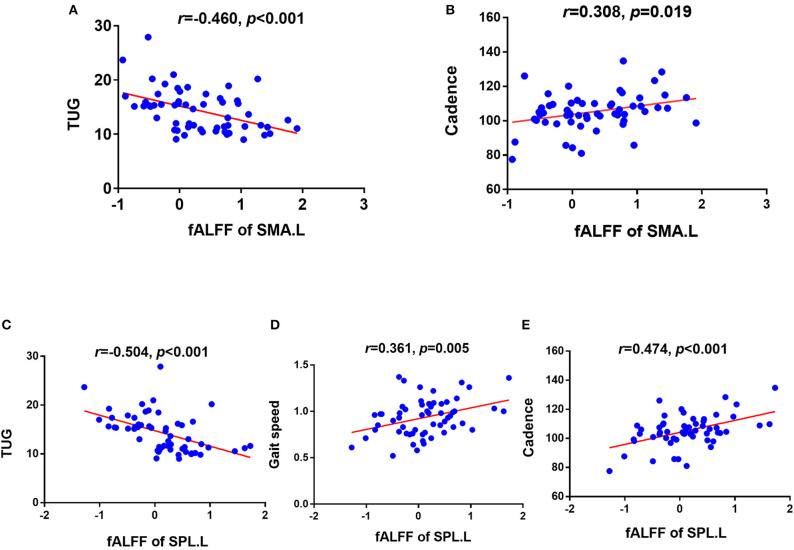
Correlation results between altered fALFF and clinical variables (*p* < 0.05, cluster-level FDR corrected). **(A)** Negative correlation between fALFF of SMA.L and TUG. **(B)** Positive correlation between fALFF of SMA.L and cadence. **(C)** Negative correlation between fALFF of SPL.L and TUG. **(D)** Positive correlation between fALFF of SPL.L and gait speed. **(E)** Positive correlation between fALFF of SPL.L and cadence. TUG, timed up and go test; SMA.L, left supplementary motor area; SPL.L, left superior parietal lobe.

The significant correlations between altered brain function value and clinical variables are illustrated graphically in Figures 4, 5 (*p* < 0.05, cluster-level FDR corrected). The strength of FC (SMA.L-LTC.R) was negatively correlated with TUG (*r* = −0.400, *p* = 0.002) (Figure 4B) and positively correlated with gait speed (*r* = 0.266, *p* = 0.043) (Figure 4C). There was negative correlation between the fALFF of SMA.L and TUG (*r* = −0.460, *p* < 0.001) (Figure 5A), and positive correlation between the fALFF of SMA.L and cadence (*r* = 0.308, *p* = 0.019) (Figure 5B). The fALFF of SPL.L was negatively correlated with TUG (*r* = −0.504, *p* < 0.001) (Figure 5C), positively associated with both gait speed (*r* = 0.361, *p* = 0.005) (Figure 5D) and cadence (*r* = 0.474, *p* < 0.001) (Figure 5E). The altered fALFF value in other regions was also negatively correlated with TUG [PoCG.R (*r* = −0.414, *p* = 0.001); IFG.R, orbital part (*r* = −0.333, *p* = 0.011), Pcu.L (*r* = −0.522, *p* < 0.001), CAU.L (*r* = −0.276, *p* = 0.036)] (*p* < 0.05, cluster-level FDR corrected). However, no significant correlations were observed between altered brain function value and neuropsychological test scores (*p* > 0.05).

## Discussion

The present study used both fALFF and seed-based FC measures to examine regional and large network brain alterations in a cohort of CSVD patients with gait disorders. Decreased and increased low-frequency oscillation amplitudes were found in multiple brain regions distributed over cortical and subcortical regions in the CSVD-GD patients. Abnormal local neural function in the SMA and its remote connectivity with the temporal lobe were observed in our study, which were associated with cadence and gait speed, respectively. The altered brain function and its significant relationship with gait may be helpful for understanding the pathophysiology and underlying neural mechanisms of gait disturbances in CSVD patients.

ALFF is a sensitive tool to detect spontaneous function in the brain. In the present study, a notable finding was that CSVD-GD patients showed significantly decreased fALFF values in the SMA region. Neuropsychological studies have suggested that the SMA is an important part of the sensorimotor network, contributing to motor processes and mediating action initiation by directly or indirectly linking to the prefrontal cortex, motor cortex, and spinal cord (Bonini et al., 2014). During movement, the SMA serves in several processes, including action preparation, action initiation and selection of actions, motor learning, inhibition, control, and monitoring roles (Lima et al., 2016). It is reasonable that the reduced local neural function in the SMA may lead to deficits in motor processes and subsequent gait disorders. A recent study exhibited significant improvements by stimulating the SMA, rather than other motor cortex or dorsal lateral prefrontal cortex, using repetitive transcranial magnetic stimulation in PD patients with freezing gait (Kim et al., 2018), which indicated the important role of SMA in the pathophysiologic processes of gait disorders.

Further FC analysis also exhibited decreased connectivity between the SMA and widespread temporal areas, such as the middle temporal lobe, the superior temporal lobe, and supramarginal gyrus. Previous studies have demonstrated that visuospatial function and vestibular information processing are related to the temporal cortex (Li et al., 2019). It is accepted that both visuospatial and vestibular function are essential for gait and balance maintenance. Structural abnormalities in the temporal lobe and supramarginal gyrus were also reported in previous studies. For instance, gray matter alterations in the superior and middle temporal gyri and supramarginal gyrus (Smith et al., 2015) and FC reductions in the medial temporal gyrus were reported in VBM and rs-fMRI studies (Wang et al., 2016). On the basis of the hypothesis that a connection exists between the temporal gyrus and SMA and that the SMA receives input from the temporal lobe, our finding of decreased FC between the SMA and widespread temporal regions sheds light on the involvement of the temporal lobe via the SMA in the neuropathology of CSVD-GD. The SMA, a pivotal motor-related hub region, was altered at both the regional and network levels in CSVD patients with gait disorders.

Compared with CSVD patients without gait disturbance, GD patients also displayed significant fALFF reductions in the PoCG and the SPL regions. The PoCG served as the hub region in somatosensory network, engaging in daily activities (Fu et al., 2019). According to previous studies, the somatosensory inputs are of great importance in regulating the ongoing movements by feed-forward adjustments (Brooks and Stoney, 1971), which is helpful for posture and gait adjudgment (Takakusaki, 2017). It was also reported that the somatosensory system dominates learning in the early stages of motor skill acquisition (Bernardi et al., 2015). Thus, the decreased integrity of somatosensory networks may suggest the reduced abilities of gait adjudgment and motor learning in patients with CSVD-GD. The SPL is a vital component of the frontoparietal network, a network closely linked to executive function (Jor'dan et al., 2017; Lo et al., 2017), which is implicated in initiating and modulating cognitive control. In previous studies, the SPL has consistently been suggested to be involved in motor coordination and control, especially when speed is decreased and variability is large (Bürki et al., 2017). Thus, the hypofunction of the SPL may be related to ineffective motor control and slow gait speed. This observation is in keeping with the findings of previous fMRI clinical studies, which found that the FC strength within the frontoparietal network was closely correlated with gait speed (Lo et al., 2017). Taken together, the altered patterns of spontaneous low-frequency oscillations in multiple regions implied that the changes in function were not confined to motor areas and that additional widespread somatosensory and frontoparietal network regions were also involved, indicating the important roles of both somatosensory inputs and control roles of cognition on motor function, which was in accordance with a previous study (Takakusaki, 2017).

A further noteworthy finding of the present study is that there were also several regions showing increased fALFF, such as the orbital part of the inferior frontal gyrus, caudate, and precuneus. The inferior frontal gyrus, orbital part, part of the orbitofrontal cortex in the prefrontal cortex, is specifically associated with cognitive processes and involved in processing the motivational or emotional value of incoming information (Rosso et al., 2017), which is suggested to be especially essential for the initiation of motor activities. The precuneus is one of the highest metabolically active brain regions in the default mode network (DMN) during the resting state that directly interacts with other nodes in the default network and participates in monitoring the external environment. Previous studies have confirmed the vital roles of the precuneus in the regulation of body movement by connecting with the precentral gyrus and SMA. The caudate nucleus also receives inputs from various prefrontal regions and plays a major part in task-switching behavior, which can be understood in terms of activating different modules for specialized function (Jarbo and Verstynen, 2015). In the current study, increased local functional connections may serve to compensate adjustments in posture and movement by enhancing the motivation process and interaction with the motor areas. Higher local brain function may act to buffer the detrimental effects of ischemic changes on slower gait.

In addition, we found that the brain FC between the SMA and temporal lobe in CSVD patients was positively related to the gait speed, whereas the fALFF in the left SMA was positively associated with cadence. Gait speed has been consistently treated as the most important factor regarding gait disorders in previous studies, which may be directly associated with incident disability, mortality, and hospitalizations (Studenski et al., 2011; Jung et al., 2018). The correlation of FC and gait speed may imply the important predication role of brain function alteration in slower gait of CSVD. Apart from the gait speed, the cadence is another essential gait parameter, which was associated with the fALFF alteration in the SMA. This was in accordance with a cortical thickness study that found that the cortical area in the SMA was positively related to cadence (de Laat et al., 2012). Based on the aforementioned findings, we suspected that the SMA may be a key region for regulating cadence. Of note, the fALFF of the left SPL was related to both the velocity and cadence in our study. These results may further emphasize the important roles of frontoparietal network on gait adjustment. In addition, we found that all the altered brain function indicators were correlated with longer time consumption of TUG, suggesting that both the local brain function alteration and network interaction abnormalities may contribute to gait disorder. To further clarify the relationship between structural and functional alterations, both total and regional GMV in the CSVD patients were examined. Relative to controls, CSVD-GD patients exhibited no significant difference in either the total GMV or local GMV based on fALFF and FC alterations. This finding of preserved structure also suggested that functional alterations may precede structural changes. However, further longitudinal studies will be helpful to examine this speculation.

Several limitations involved in this study should be considered. First, although ALFF and FC are promising tools for detecting spontaneous functional activity in the brain, several factors may influence the interpretation of our results, for example, low signal-to-noise ratio of the acquired neuroimaging data, poor temporal resolution, and potential confounding effects on neurovascular coupling (such as concomitant vascular diseases). Thus, we should take caution when interpreting our findings. Second, the sample size was relatively small, which limits the statistical power; thus, the findings of the present study should be considered preliminary. Further study should be carried out to verify the results in the future. Third, the present study was a cross-sectional design, and whether these abnormalities in local brain function and remote connectivity dynamically change should be explored with a follow-up study. In addition, all patients included in the study had no severe muscle power or ankle problems, but we did not explicitly evaluate these factors. Finally, the healthy elderly were not included in the present study, and the differences in mechanism of gait disorder were not further explored in CSVD with different levels of cognitive impairment; classifying and stratifying patients in future studies are needed to extend the understanding of the pathogenesis of gait disorders in CSVD.

## Conclusion

In the current study, we investigated the possible pathogenesis of CSVD-GD by analyzing resting-state local brain function and large distance connectivity patterns in CSVD patients. The results suggested that abnormal changes in spontaneous function and remote connectivity in the sensorimotor network, frontoparietal network, and DMN may explain gait disorders, instability, and aggravating factors in CSVD patients. These findings might provide novel insights into pathophysiological mechanisms in gait disturbances.

## Data Availability Statement

All datasets generated for this study are included in the article/supplementary material.

## Ethics Statement

The studies involving human participants were reviewed and approved by the Institutional Review Board of the first affiliated hospital of Anhui Medical University Subcommittee on Human Studies. The patients/participants provided their written informed consent to participate in this study.

## Author Contributions

XZho, CZ, and ZS conceived and designed the experiments. LL, YZ, XY, WY, WZ, XZhu, and YQ performed the experiments. XZho, CZ, and YQ analyzed the data and created the figures. XZho and ZS wrote the article and provided the funding. All authors approved the final version of the manuscript.

## Conflict of Interest

The authors declare that the research was conducted in the absence of any commercial or financial relationships that could be construed as a potential conflict of interest.
